# Vitrification of particulated articular cartilage via calculated protocols

**DOI:** 10.1038/s41536-021-00123-5

**Published:** 2021-03-19

**Authors:** Kezhou Wu, Nadia Shardt, Leila Laouar, Janet A. W. Elliott, Nadr M. Jomha

**Affiliations:** 1 Department of Surgery, University of Alberta, Edmonton, AB Canada; 2 Department of Orthopedic Surgery, First Affiliated Hospital, Shantou University Medical College, Shantou, Guangdong China; 3 Department of Chemical and Materials Engineering, University of Alberta, Edmonton, AB Canada; 4 Department of Laboratory Medicine and Pathology, University of Alberta, Edmonton, AB Canada

**Keywords:** Tissues, Regenerative medicine, Tissue engineering

## Abstract

Preserving viable articular cartilage is a promising approach to address the shortage of graft tissue and enable the clinical repair of articular cartilage defects in articulating joints, such as the knee, ankle, and hip. In this study, we developed two 2-step, dual-temperature, multicryoprotectant loading protocols to cryopreserve particulated articular cartilage (cubes ~1 mm^3^ in size) using a mathematical approach, and we experimentally measured chondrocyte viability, metabolic activity, cell migration, and matrix productivity after implementing the designed loading protocols, vitrification, and warming. We demonstrated that porcine and human articular cartilage cubes can be successfully vitrified and rewarmed, maintaining high cell viability and excellent cellular function. The vitrified particulated articular cartilage was stored for a period of 6 months with no significant deterioration in chondrocyte viability and functionality. Our approach enables high-quality long-term storage of viable articular cartilage that can alleviate the shortage of grafts for use in clinically repairing articular cartilage defects.

## Introduction

Articular cartilage defects are difficult to self-repair due to the avascular and aneural structure of articular cartilage^1^. Particulated articular cartilage transplantation can provide viable chondrocytes in situ for articular cartilage repair, and it is regarded as a reliable treatment for articular cartilage defects in the early stage, especially for focal chondral lesions^2–4^. Since 2007, ~8700 particulated articular cartilage transplantations have been performed and significant improvement was reported in the clinical outcomes of patients with articular cartilage defects^5^. However, this procedure is limited by the availability of donated articular cartilage. Fresh articular cartilage is most commonly stored for up to 28 days at 4 °C in a tissue bank, and chondrocyte viability starts to decline after 14 days of storage at this temperature^6,7^. This short window of time for viable articular cartilage makes successful cartilage transplantation challenging in the clinical scenario, because it takes ~2 weeks to obtain regulatory clearance for disease screening and make clinical preparations such as patient matching, disease checking, operation scheduling, and sample delivery^6,8^. The lack of appropriate preservation protocols for storage results in large amounts of articular cartilage waste every year around the world^8^. If viable articular cartilage could be stored for extended periods of time beyond 28 days, the shortage of articular cartilage tissue in clinics would be eased, and more widespread clinical use of articular cartilage grafts (e.g., particulated articular cartilage) for cartilage defect repair would be possible.

Preservation of articular cartilage has been a challenge for decades, especially for large specimens (e.g., osteochondral grafts, hemicondyles) containing live cells that are very sensitive to osmotic stress, chemical toxicity, and ice growth during the cooling and warming processes^9–11^, and this sensitivity compromises cell survival and function. Cryopreservation by vitrification is an ice-free storage method that can be achieved by superfast cooling or in the presence of high concentrations of cryoprotectants to inhibit ice crystallization inside the tissue^12^. Porcine articular cartilage has been used as a successful animal model for articular cartilage research due to its biological similarity to human articular cartilage, especially related to its thickness in the hind stifle joint relative to the human knee joint^9,13^. Past work in the vitrification of articular cartilage tissue has investigated the use of single or multicryoprotectant solutions at one or several progressively decreasing temperatures: Pegg et al.^14^ loaded dimethyl sulfoxide (DMSO) into thin lamb articular cartilage slices at progressively lower temperatures that traced the liquidus line of the solid–liquid phase diagram to a sufficiently high concentration of cryoprotectants inside the cartilage before storage at −80 °C; Brockbank et al.^15,16^ loaded multiple cryoprotectants (DMSO, formamide, propylene glycol (PG)) into porcine articular cartilage plugs (6 mm diameter osteochondral plugs) gradually from low-to-high concentrations at 4 °C before rapid cooling to −135 °C; and our group developed a stepwise loading method that loads multiple cryoprotectants (DMSO, ethylene glycol (EG), PG, glycerol) at progressively lower temperatures from 0 °C down to −20 °C to reach a high concentration of cryoprotectants within human articular cartilage dowels (10 mm diameter osteochondral plugs and 12.5 cm^2^ large fragments) before plunging into liquid nitrogen at −196 °C for vitrification^13^.

Given the practically inaccessible numbers of potential cryoprotectant combinations, temperatures, and times of exposure, mathematical modeling provides a systematic framework for screening and identifying successful cryoprotectant loading protocols. Such models can make calculations of pertinent properties—cryoprotectant concentration, cytotoxicity, solution freezing point, vitrifiability, and/or temperature—as a function of location in the cartilage and time of exposure. For example, cryoprotectant concentration has been calculated using biomechanical models^17–19^ and Fick’s law^20–23^; a mathematical toxicity cost function was introduced by Benson et al.^24^ to quantify the cumulative toxicity experienced by cells over the course of cryoprotectant loading; and vitrifiability^23,25^ and freezing point^23,26,27^ have been accurately modeled. These mathematical models usually require parameters extracted from experimental data that can depend on tissue type, cryoprotectant type, and temperature.

To implement mathematical models for articular cartilage preservation, the kinetic diffusion parameters in porcine articular cartilage, osmotic virial coefficients, and contributions to multicryoprotectant solution vitrifiability of three common cryoprotectants—DMSO, EG, and PG—have previously been obtained by our group: Abazari et al.^28^ determined parameters for the temperature-dependent diffusion kinetics of these cryoprotectants in porcine articular cartilage by fitting Fick’s law of diffusion to experimental measurements (using these empirically fitted diffusion coefficients for kinetic predictions of cryoprotectant diffusion has been shown to agree reasonably with predictions made using the nonideal triphasic biomechanical model that considers water transport^19^ and with experimental measurements of efflux from human cartilage^22^); Zielinski et al.^26^ determined osmotic virial coefficients for common cryoprotectants by fitting the multisolute osmotic virial equation proposed by Elliott et al.^26,27,29,30^ to experimental data of freezing point; and Weiss et al.^25^ quantified the vitrifiability of multicryoprotectant saline solutions using a statistical model fit to experimental observations of 5 mL cryoprotectant solutions with concentrations between 6 and 9 M placed in 10 mL polypropylene tubes and plunged in liquid nitrogen (a ~60 K/min cooling rate^25,31^) for 30 min, followed by warming in a 37 °C water bath^25^. Our first successful vitrification protocol^13^ was developed for osteochondral dowels (articular cartilage with underlying bone), where each of four cryoprotectants was loaded sequentially in a separate loading step to attain a specified minimum concentration of each cryoprotectant at the cartilage–bone junction over a total loading time of 9.5 h. Then, we investigated the diffusion dynamics of this 2012 protocol, proposing a hypothetical 3-step loading procedure totaling 7 h in duration that still attained the targeted minimum concentration of each cryoprotectant^22^. Based on the work by Shardt et al.^23^, we further modified the 2012 protocol by (i) reducing all cryoprotectant loading solution concentrations, (ii) removing glycerol as a cryoprotectant, and (iii) introducing an equilibration step. In addition, we calculated the spatial and temporal distribution of vitrifiability, and based on this calculation, we designated potential loading protocols as having sufficient cryoprotectant permeation once the minimum vitrifiability exceeded the threshold determined by Weiss et al.^25^ arriving at a more optimal 7 h protocol^23^ for osteochondral dowels shown experimentally to be successful at preserving chondrocyte viability and metabolism in porcine articular cartilage^32^.

Herein, we present two 2-step, dual-temperature, multicryoprotectant protocols for vitrifying particulated articular cartilage (cubes ~1 mm^3^ in size) for extended periods of time (6 months) at cryogenic temperatures while maintaining high cell viability and function for use in a clinical setting. We use mathematical modeling to determine optimal cryoprotectant exposure times and temperatures (via calculations of spatially and temporally resolved cryoprotectant concentration, solution freezing point, and vitrifiability, while qualitatively minimizing cytotoxicity with an appropriate selection of cryoprotectant type and concentration in the loading solution and not unnecessarily exceeding required loading times). We prove the success of these protocols by experimentally measuring chondrocyte viability, metabolic activity, cell migration, and matrix productivity after cryoprotectant loading, vitrification, and rewarming of both porcine and human particulated articular cartilage. Pigs are considered the best animal model for articular cartilage cryopreservation^33^; thus, we include porcine samples to show the best case scientific results when tissue is harvested immediately post mortem from young individuals compared with the more clinically applicable results for human where tissue is harvested from deceased older donors some hours after death.

## Results

### Computational generation of cryoprotectant loading protocols for particulated articular cartilage

We develop optimized protocols based on a combined consideration of the spatial and temporal distribution of cryoprotectant concentration, solution freezing point, and cryoprotectant vitrifiability, an approach that we used for the development of successful protocols for preserving articular cartilage dowels^13,23,32^. Based on the toxicity studies of Almansoori et al.^34^ and Jomha et al.^35^ that ranked the relative toxicity of commonly used cryoprotectants for human and porcine chondrocytes, we selected the least toxic compounds for our protocols: EG, DMSO, and PG. Our first protocol loaded all three cryoprotectants (named Protocol E–D–P), and the second protocol loaded only two cryoprotectants (EG and DMSO; named Protocol E–D) because out of the three cryoprotectants, PG is associated with the most cytotoxicity^36^.

**Fig. 1:**
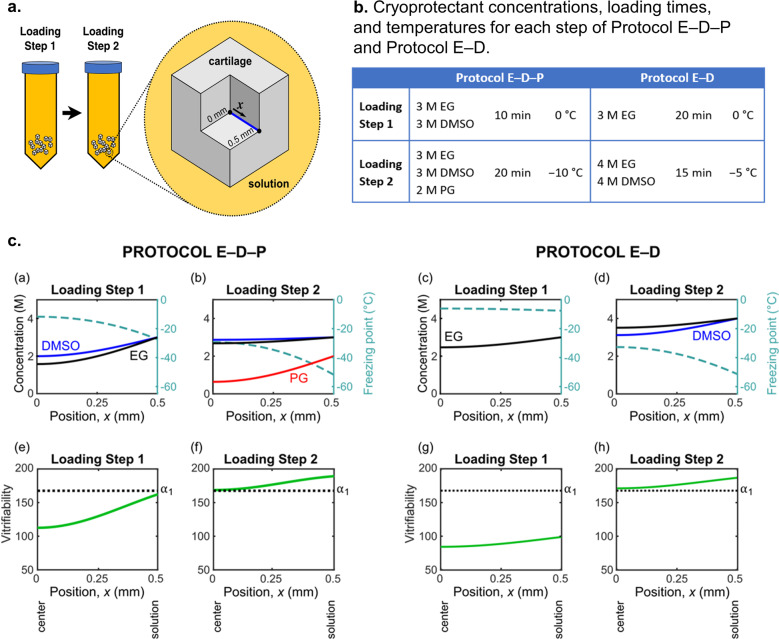
Overview of theoretical calculations. **a** Schematic of the 1 mm^3^ articular cartilage cubes. For our theoretical calculations, we consider a one-dimensional line for diffusion with length 0.5 mm extending from the center of the cube to the edge of the cube that is in contact with the cryoprotectant solution. **b** Summary of cryoprotectant concentrations, loading times, and temperatures for each step of Protocol E–D–P and Protocol E–D. Each protocol consists of two steps, with the second step introducing an additional cryoprotectant at a reduced temperature. **c** Calculations of the spatial distribution of cryoprotectant concentration, solution freezing point, and vitrifiability at the end of each loading step. For these calculations, we used Fick’s law of diffusion, the multisolute osmotic virial equation combined with the Gibbs–Duhem equation^26,27,30^, and a statistical model of vitrifiability^25^. In (e)–(h), *α*_1_ indicates the minimum vitrifiability score required for vitrification under the experimental conditions described in Weiss et al.^25^.

To predict spatial and temporal distributions of concentration, we used Fick’s law of diffusion over a one-dimensional line through a cube of cartilage (Fig. 1a) with effective diffusion coefficients previously obtained by fitting to experiments that measured the uptake of cryoprotectant in porcine cartilage as a function of time and temperature^28,37^. We note that Fick’s law underestimates cryoprotectant diffusion when compared to experimental measurements and to our group’s nonideal triphasic model^19^, but it is within an average of 15% from experimental measurements of cryoprotectant efflux from human cartilage dowels (using Fick’s law in two dimensions with diffusion coefficients from porcine cartilage as an approximation of those in human)^22^. Thus, we used Fick’s law in one dimension to ensure that we developed conservative protocols that will be robust to small variations in, for example, cube size, diffusion coefficients (inhomogeneities in the cartilage matrix may cause deviations), and heat transfer rates during vitrification (in our experiments, we achieve a cooling rate of ~140 K/min, where 0.5 g of articular cartilage cubes after cryoprotectant permeation are placed in a dry 1.8 mL Cryovial and plunged into liquid nitrogen, reaching −150 °C from −10 °C in ~60 s based on five independent measurements).

To predict spatial and temporal distributions of freezing point accounting for the nonideal behavior of highly concentrated cryoprotectant solutions, we used the multisolute osmotic virial equation^26,27,29,30^ with the osmotic virial coefficients determined by Zielinski et al.^26^ combined with the Gibbs–Duhem equation^26,27,30^. To predict spatial and temporal distributions of vitrifiability, we used the statistical model developed by Weiss et al.^25^. Based on the criterion of achieving a minimum level of vitrifiability throughout the cube^23^, we developed two new protocols (Protocol E–D–P and Protocol E–D) for 1 mm^3^ particulated cartilage cubes, as summarized in Fig. 1b, with more details of the design process outlined in the “Methods” section. Figure 1c shows the calculated spatial distribution of cryoprotectant concentration, freezing point, and vitrifiability at the end of each loading step for Protocol E–D–P and Protocol E–D. By the end of the second loading step, both protocols (Tables 1 and 2) are predicted to exceed the minimum vitrifiability threshold (*α*_1_ = 167.6) at all locations in the cartilage from the center of the cube to the edge in contact with the cryoprotectant solution.

**Table 1:** Minimum cryoprotectant concentrations and vitrifiability scores and maximum freezing point calculated at the end of each loading step in Protocol E–D–P (located at the center of the cartilage cube where x = 0 mm).

Protocol E–D–P	Minimum concentrations	Minimum vitrifiability score	Maximum freezing point
Loading step 1	1.57 M EG 1.99 M DMSO	113	−12 °C
Loading step 2	2.68 M EG 2.87 M DMSO 0.64 M PG	169	−29 °C

**Table 2:** Minimum cryoprotectant concentrations and vitrifiability scores and maximum freezing point calculated at the end of each loading step in Protocol E–D (located at the center of the cartilage cube where x = 0 mm).

Protocol E–D	Minimum concentrations	Minimum vitrifiability score	Maximum freezing point
Loading step 1	2.47 M EG	84	−6 °C
Loading step 2	3.51 M EG 3.12 M DMSO	171	−33 °C

### Experimental vitrification of articular cartilage cubes

**Fig. 2:**
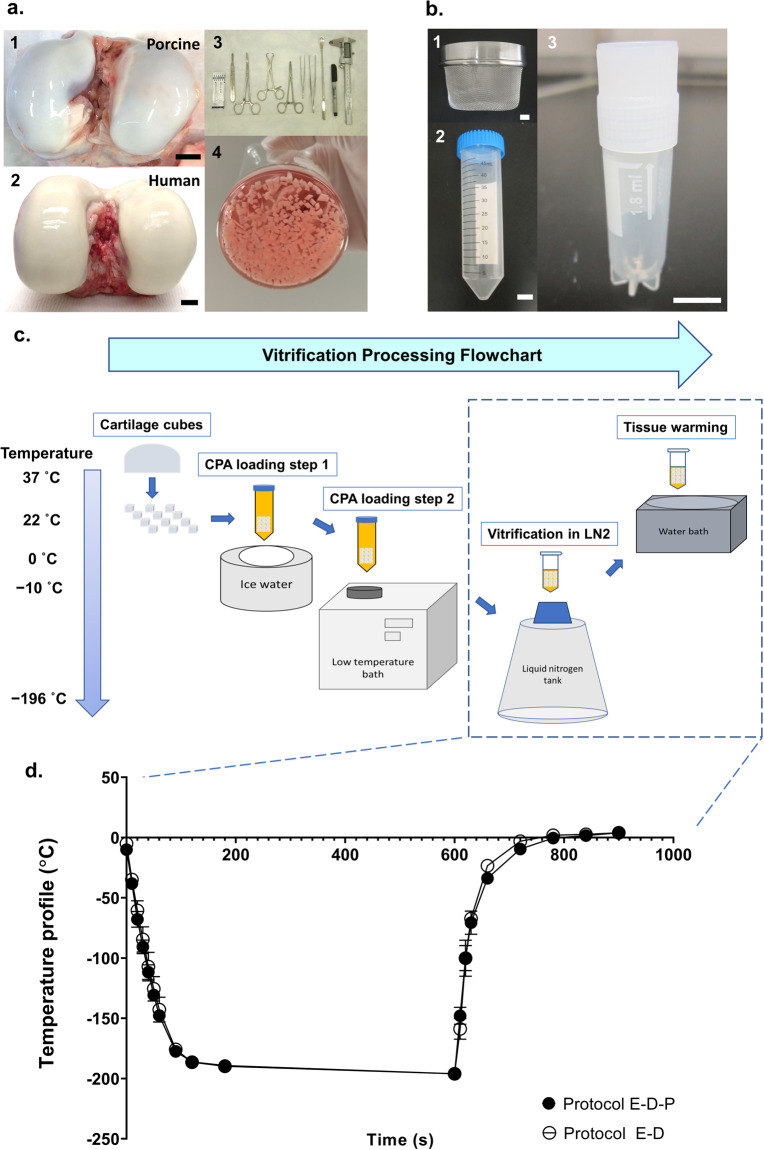
Vitrification process of particulated articular cartilage. **a** Harvesting articular cartilage cubes from fresh healthy knee joints. 1 A typical porcine stifle joint with exposed fresh healthy femoral condyles. 2 A typical human knee with exposed fresh healthy femoral condyles. 3 The surgical tool set, from left to right: #20 steel surgical blade, surgical scalpel handle, curved forceps, towel forceps, straight forceps, non-toothed tissue forceps, toothed tissue forceps, double-ended spoon, Sharpie marker, and digital caliper. 4 Cartilage prepared into 1 mm^3^ pieces and placed in medium before vitrification. **b** The vitrification tool set. 1 Mesh strainer used for transferring cartilage cubes. 2 The 50 mL Falcon tube for cryoprotectant loading into articular cartilage. 3 1.8 mL Cryovial for sample vitrification and storage in liquid nitrogen. **c** Flowchart of the vitrification process for articular cartilage. Weighed cartilage cubes were transferred into a 50 mL prepared multicryoprotectant solution at the concentration, time, and temperature listed in Fig. 1b under loading step 1, followed by a second solution at the condition listed under loading step 2 in Fig. 1b. Once cryoprotectant permeation was finished, cartilage cubes were quickly transferred into a 1.8 mL Cryovial tube and plunged into liquid nitrogen on a Cryovial cane. Upon cartilage rewarming on day 0 or on day 180, the Cryovial tube was removed from the liquid nitrogen and plunged into a 37 °C water bath for warming the glassy cartilage, following by cryoprotectant removal and chondrocyte assessments. **d** Temperature profile. The thermal history of the vitrification and rewarming processes of Protocol E–D–P and Protocol E–D (*n* = 5 replicates per group). Error bars = SD. All data are represented as mean ± SD. Scale bar = 10 mm.

Based on the modeling results (Fig. 1) for cryoprotectant permeation into particulated articular cartilage, we developed the processing protocol to vitrify, store, and warm particulated articular cartilage, illustrated in Fig. 2. Briefly, after harvesting articular cartilage from healthy femoral condyles, it is sliced into 1 mm^3^ cubes under sterile conditions (Fig. 2a). Approximately 0.5–0.8 g wet weight of particulated articular cartilage per protocol treatment was loaded in 50 mL of multicryoprotectant cocktail solution in 50-mL Falcon tubes. Then, two 2-step cryoprotectant loading protocols (Protocol E–D–P and Protocol E–D in Fig. 1b) with calculated loading times and preset temperatures at each step were followed to load sufficient amounts of cryoprotectants into the cartilage cubes. After the cryoprotectant permeation procedure, the particulated cartilage cubes were quickly removed from the Falcon tubes with the mesh strainer and transferred into a sterile 1.8 mL Cryovial tube with a residual amount of cryoprotectants remaining with the cartilage cubes, then plunged into liquid nitrogen on a Cryovial cane (Fig. 2b). After storage in liquid nitrogen at −196 °C for the desired experimental time period (one tube warmed at day 0 and one tube warmed at day 180), the Cryovial tube containing vitrified cartilage cubes was quickly removed from the liquid nitrogen and rewarmed in a 37 °C water bath until the glass was melted, which took ~30 s for warming (Fig. 2c). Figure 2d shows the thermal history of the vitrification and rewarming processes of Protocol E–D–P and Protocol E–D. The cartilage cubes were extracted with a sterile lab spoon and washed in a beaker filled with 25 mL fresh medium (see the “Methods” section) to remove the cryoprotectants at 4 °C for 30 min and the wash was repeated three times before chondrocyte assessments.

### Assessment of chondrocytes after vitrification/tissue rewarming

After vitrifying and rewarming porcine and human particulated cartilage, we assessed the viability and function of chondrocytes both in situ in intact cartilage and after isolation from the cartilage matrix. We quantified chondrocyte membrane integrity, metabolic activity, migration ability, and matrix productivity.

**Fig. 3:**
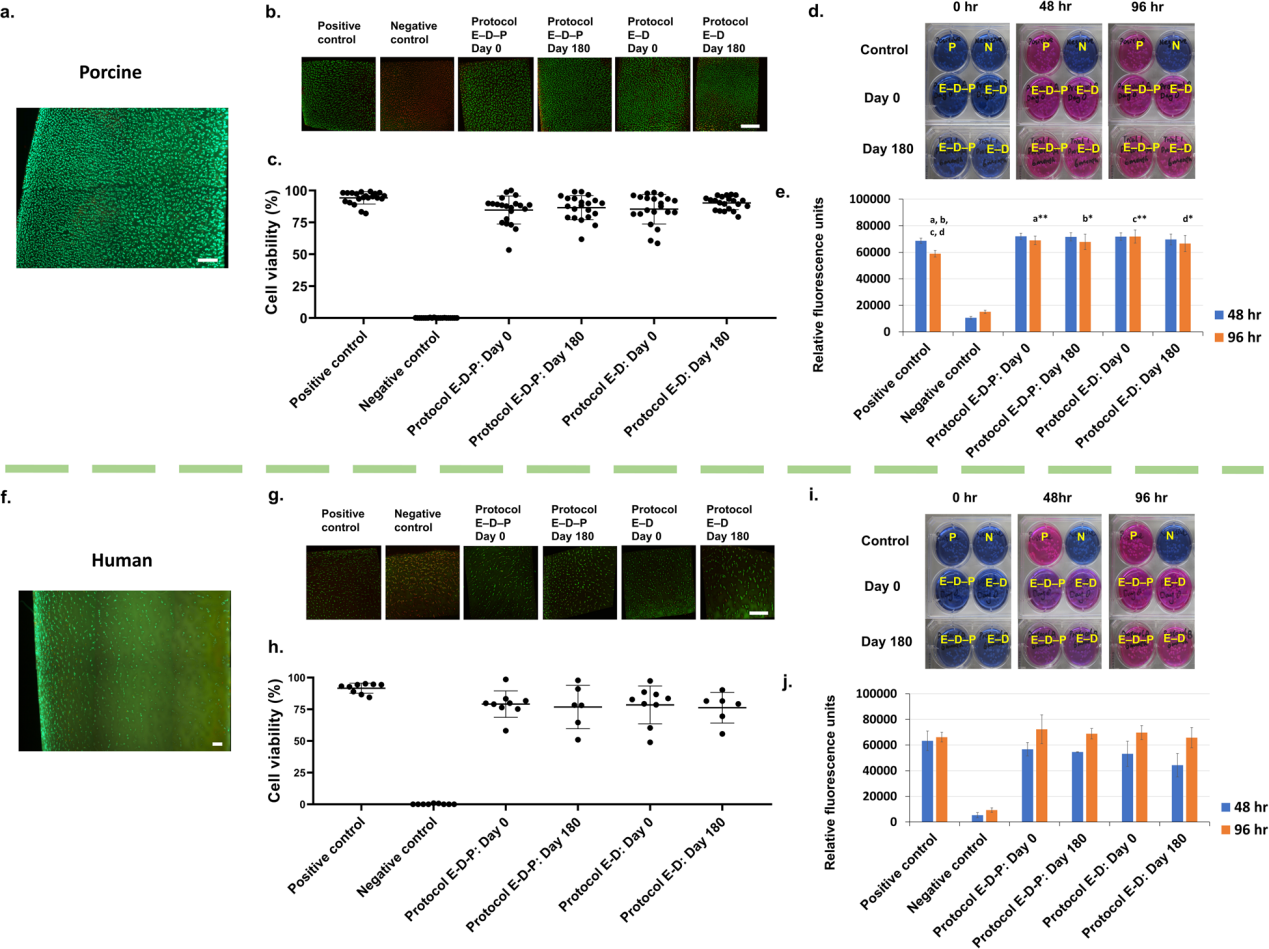
Assessment of cell viability and metabolic function of chondrocytes in situ after vitrification and subsequent rewarming. **a** Representative image of healthy full-thickness porcine cartilage section. **b** Representative images of porcine cartilage samples showing live (green) and dead (red) chondrocytes by a cell membrane integrity stain using two fluorescent dyes: Syto 13 and propidium iodide and imaged by a laser scanning confocal microscope (×100 magnification). **c** Quantification of porcine chondrocyte viability of the positive control, negative control, Protocol E–D–P at day 0, Protocol E–D–P at day 180, Protocol E–D at day 0, and Protocol E–D at day 180 (*n* = 21 replicates per group: no statistical differences between the protocols and the storage times, *p* = 0.261, two-way ANOVA). **d** Representative images of the alamarBlue assay showing porcine chondrocyte metabolic activity from particulated articular cartilage in positive (P) and negative (N) controls and after vitrification at 0, 48, and 96 h. **e** Quantification of porcine chondrocyte metabolic activity (*n* = 7 replicates per group: significant rebound of chondrocyte metabolic activity at 96 h in the experimental groups compared to the positive control, *p*_a_ = 0.003; *p*_b_ = 0.011; *p*_c_ < 0.001; *p*_d_ = 0.035, two-way ANOVA and Tukey’s test). **f** Representative image of healthy full-thickness human cartilage section. **g** Representative images of human cartilage samples showing live (green) and dead (red) chondrocytes by a cell membrane integrity stain using two fluorescent dyes: Syto 13 and propidium iodide and imaged by a laser scanning confocal microscope (×100 magnification). **h** Quantification of human chondrocyte viability of the positive control, negative control, Protocol E–D–P at day 0, Protocol E–D–P at day 180, Protocol E–D at day 0, and Protocol E–D at day 180 (*n* = 6–9 replicates per group: no statistical differences between the protocols and the storage times, *p* = 0.706, two-way ANOVA). **i** Representative images of the alamarBlue assay showing human chondrocyte metabolic activity from particulated articular cartilage in positive (P) and negative (N) controls and after vitrification at 0, 48, and 96 h. **j** Quantification of human chondrocyte metabolic activity with a similar level comparing the experimental groups to the positive control (*n* = 3 replicates per group, *p* = 0.823, based on two-way ANOVA) and a hypermetabolic rebound between 48 and 96 h in both Protocol E–D–P and Protocol E–D groups. Error bars = SD. All data are represented as mean ± SD. For statistical differences, *p < 0.05, **p < 0.01. Scale bar = 200 µm.

Maintaining high chondrocyte viability after storage is vital for articular cartilage transplantation, where cell viability greater than 70% facilitates improved outcomes in the clinical scenario^7,38^. To evaluate chondrocyte viability after our proposed vitrification protocols, we used fluorescent dyes (Syto 13 and propidium iodide) to quantify the percentages of chondrocytes with intact or disrupted membranes. For porcine particulated articular cartilage (Fig. 3a, b), the fresh positive control group had an absolute cell membrane integrity of 94.2 ± 4.8% (mean ± standard deviation (SD)), and the experimental groups treated with either Protocol E–D–P or Protocol E–D had 85–90% cell membrane integrity (Fig. 3c, see Supplementary Table 1 for numeric data). After vitrification and warming, ~90% normalized chondrocyte membrane integrities were maintained in both Protocol E–D–P and Protocol E–D groups. Importantly, there were no statistically significant differences in chondrocyte viability between Protocol E–D–P and Protocol E–D groups after vitrification and warming at day 0. After 6 months of storage in liquid nitrogen, the chondrocyte viability of porcine particulated articular cartilage remained as high as that vitrified and warmed at day 0 using either Protocol E–D–P or Protocol E–D (Fig. 3c).

For human particulated articular cartilage (Fig. 3f, g), the absolute chondrocyte viability of the fresh positive control group was 91.6 ± 4.0%. After vitrification and warming, greater than 80% normalized chondrocyte membrane integrities were maintained in both Protocol E–D–P and Protocol E–D groups (Fig. 3h, see Supplementary Table 2 for numeric data). There were no statistically significant differences in the absolute chondrocyte viability between Protocol E–D–P and Protocol E–D groups at day 0. Storage of human particulated articular cartilage for 6 months in liquid nitrogen showed no statistically significant differences using either Protocol E–D–P or Protocol E–D when compared to the particulated articular cartilage that had been vitrified and warmed at day 0 (Fig. 3h).

A defining function of viable chondrocytes is their metabolic activity associated with various physiologic pathways within the normal cellular contents. We used alamarBlue to determine metabolic activity of viable chondrocytes indicated by a change from nonfluorescent resazurin (blue color) to highly fluorescent resorufin (red color) through the reduction reaction of active living cells^39,40^. Porcine particulated articular cartilage vitrified using either Protocol E–D–P or Protocol E–D and warmed at day 0 demonstrated a similar metabolic activity to the fresh positive control after 48 h, and showed a higher cell metabolic activity than the positive control after 96 h (Fig. 3d, e, see Supplementary Table 3 for numeric data). These findings are consistent with our recent work^13,32^, which showed articular cartilage that maintained high chondrocyte viability after vitrification had a level of chondrocyte metabolic activity that was activated or enhanced upon sample warming, possibly due to some sort of hypermetabolic rebound from cryogenic temperatures accompanied with mitochondrial repair inside the postvitrified chondrocytes. A confirmatory low chondrocyte metabolic activity was observed in the negative control which remained blue in color for both 48 and 96 h time points (Fig. 3e). Similarly, human particulated articular cartilage vitrified using either Protocol E–D–P or Protocol E–D and warmed at day 0 demonstrated similar metabolic activity to the fresh positive control after 48 h or after 96 h incubation (Fig. 3i, j, see Supplementary Table 4 for numeric data). A similar hypermetabolic rebound was observed in the postvitrified human chondrocytes using either Protocol E–D–P or Protocol E–D and warmed after 6 months (Fig. 3j). The alamarBlue results showed that both porcine and human particulated articular cartilage vitrified in liquid nitrogen and warmed after 6 months maintained a high level of metabolic activity comparable to the articular cartilage vitrified in liquid nitrogen and warmed at day 0 and to fresh positive controls.

**Fig. 4:**
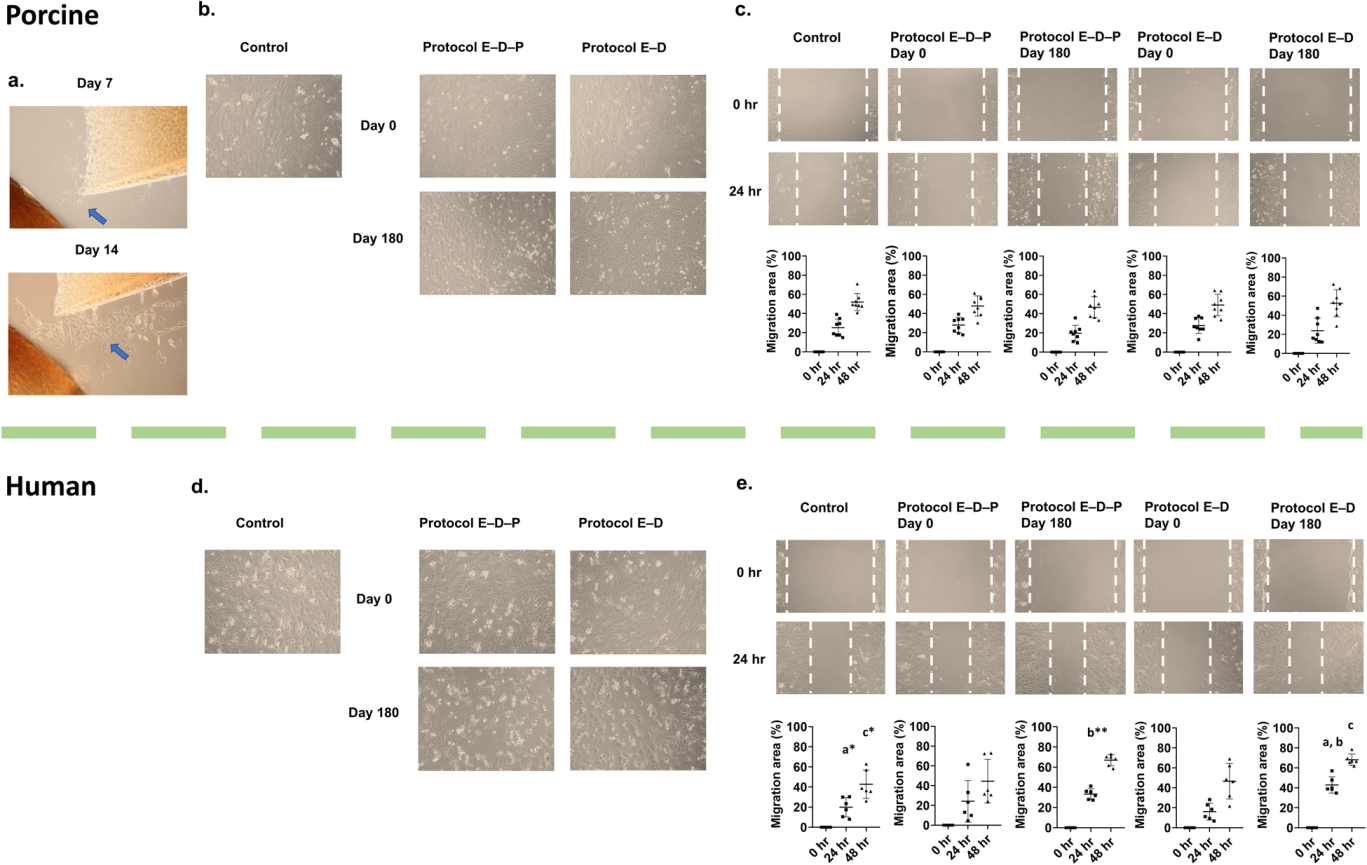
Assessment of cell migrating ability of chondrocytes isolated from articular cartilage after vitrification and subsequent rewarming. **a** Active viable porcine chondrocytes from particulated articular cartilage migrated from the cartilage matrix and proliferated in in vitro culture at day 7 and day 14. **b** Isolated porcine chondrocytes from post-vitrified articular cartilage dedifferentiated into fibroblast-like cells and migrated to the center of the culture plates (×100 magnification). **c** Isolated porcine chondrocytes from fresh control and vitrified particulated articular cartilage migrated at similar speeds to heal the scratch wound when cultured in vitro (*n* = 8 replicates per group: *p* = 0.811, two-way ANOVA). **d** Isolated human chondrocytes from postvitrified articular cartilage dedifferentiated into fibroblast-like cells and migrated to the center of the culture plates (×100 magnification). **e** When samples were stored for 6 months, isolated human chondrocytes from particulated articular cartilage using Protocol E–D migrated to heal the scratch wound with a faster migrating speed than the positive control, and the Protocol E–D group showed a faster migration than the Protocol E–D–P group at 24 h when cultured in vitro (*n* = 6 replicates per group, *p*_a_ = 0.016, *p*_b_ = 0.004, *p*_c_ = 0.043, two-way ANOVA with Tukey’s test). Error bars = SD. All data are represented as mean ± SD. For statistical differences, *p  < 0.05, **p < 0.01.

In order for articular cartilage repair to occur after implantation, chondrocytes must maintain their ability to migrate from the cartilage matrix and proliferate^2,3^. An in vitro culture of porcine particulated articular cartilage showed chondrocytes migrating from the cartilage matrix between two cartilage cubes (Fig. 4a). This observation demonstrated that chondrocytes maintained good cellular function, an important characteristic for orthopedic surgeons to consider in the clinical scenario^2^. To further confirm that chondrocytes from vitrified articular cartilage maintained a high functionality, we evaluated the ability of chondrocytes isolated from the cartilage matrix to proliferate in vitro. Isolated chondrocytes from articular cartilage cubes were seeded on a 24-well culture plate and grown with culture medium as described in the “Methods” section. Both porcine (Fig. 4b) and human (Fig. 4d) chondrocytes isolated after vitrification using either Protocol E–D–P or Protocol E–D demonstrated a similarly strong proliferating ability to become confluent in the culture plates after 168 h. To further investigate the migration ability of chondrocytes, a 1-mm wide section of expanded cells was removed by scratching and then followed by another 48-h cell culture in the incubator, during which time the viable and active chondrocytes migrated to fill the gap. The results confirmed that our vitrification approaches for articular cartilage storage up to 6 months do not impair chondrocyte migration ability as shown in Fig. 4c, e.

**Fig. 5:**
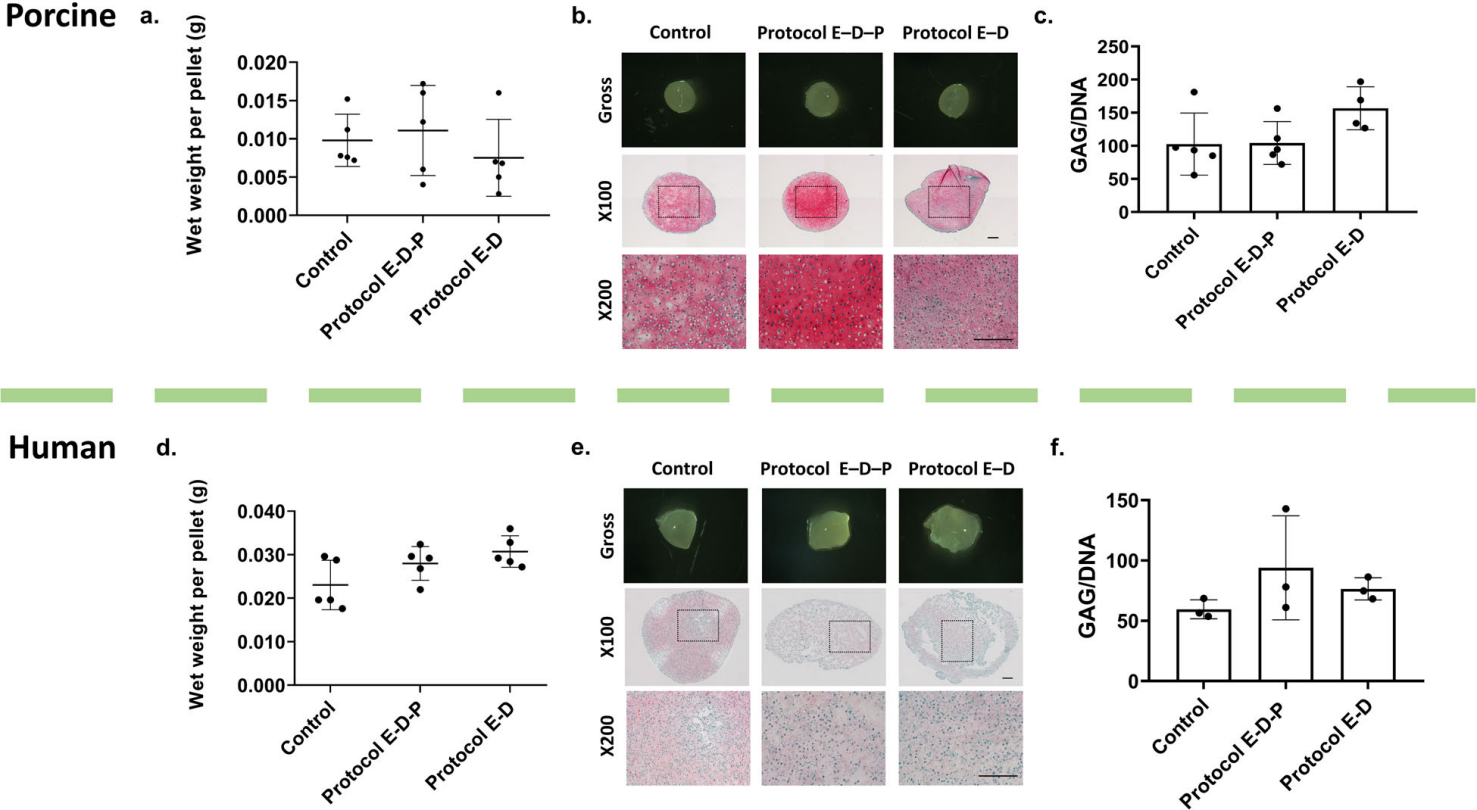
Assessment of matrix productivity of chondrocytes isolated from articular cartilage after vitrification and subsequent rewarming. **a** Porcine pellets after a 21-day culture show a similar wet weight among the fresh control, Protocol E–D–P, and Protocol E–D groups (*n* = 5 replicates per group, *p* = 0.074, one-way ANOVA). **b** Representative histological images showing the gross appearance of porcine pellets after a 21-day culture with chondrogenic media under hypoxic conditions (3% O_2_, 5% CO_2_ at 37 °C). Safranin O stain showing GAG content synthesized by viable and active chondrocytes for the fresh control, Protocol E–D–P, and Protocol E–D groups. **c** GAG/DNA quantification of porcine pellets show similar matrix productivity among the fresh control, Protocol E–D–P, and Protocol E–D groups (*n* = 4–5 replicates per group, *p* = 0.106, one-way ANOVA). **d** Human pellets after a 21-day culture show similar wet weights among the fresh control, Protocol E–D–P, and Protocol E–D groups (*n* = 5 replicates per group, *p* = 0.054, one-way ANOVA). **e** Representative histological images showing the gross appearance of human pellets after a 21-day culture with chondrogenic media under hypoxic conditions (3% O_2_, 5% CO_2_ at 37 °C). Safranin O stain showing GAG content synthesized by viable and active chondrocytes for the fresh control, Protocol E–D–P, and Protocol E–D groups. **f** GAG/DNA quantification of human pellets show similar matrix productivity among the fresh control, Protocol E–D–P, and Protocol E–D groups (*n* = 3 replicates per group, *p* = 0.334, one-way ANOVA). Error bars = SD. All data are represented as mean ± SD. Scale bar = 200 µm.

The properties of the articular cartilage matrix generated by chondrocytes are important criteria to evaluate the functionality of viable chondrocytes. First, after culture for 21 days, we compared the wet weight of the cultured pellets to represent the amount of extracellular matrix produced. For both the porcine (Fig. 5a) and human (Fig. 5d) chondrocyte pellets, there were no significant differences between the fresh positive control, Protocol E–D–P, and Protocol E–D groups. Secondly, Fig. 5b, e shows the gross morphology of chondrocyte pellets with pink-colored Safranin O staining for sulfated glycosaminoglycans (GAGs) of cartilage sections after the 21-day culture period. GAG production is essential in maintaining the integrity of normal cartilage. A similar pink color was observed for the fresh positive control groups and the vitrified groups: Protocol E–D–P and Protocol E–D. Thirdly, the amount of GAG per DNA is an important parameter to evaluate the chondrocyte synthesis functionality. To document this, we quantified the GAG content using a dimethylmethylene blue (DMMB) assay to detect the amount of GAG being produced by postvitrified chondrocytes, and the DNA content in the pellets using a DNA kit (see the “Methods” section). There were no statistically significant differences in the GAG/DNA content between the fresh positive control, Protocol E–D–P, and Protocol E–D groups for both porcine (Fig. 5c) and human (Fig. 5f) chondrocyte pellets.

## Discussion

In this study, we applied our mathematical modeling to generate new protocols and we used these protocols to successfully vitrify and store particulated articular cartilage at cryogenic temperature, filling the gap in the current literature for protocols that can vitrify a size between osteochondral allografts with a bone base and isolated chondrocytes digested from cartilage fragments. Our approach lengthens the time available for clinical transplantation to 6 months, compared to the current standard of 28 days^7^, providing an extended period of time for arranging surgical procedures. This 6-month storage time will alleviate the shortage of grafts and thus permit a larger number of particulated articular cartilage transplantations to take place, which is a clinically significant advance. Theoretically, storage as proposed in this paper can be indefinite without any expected change in results due to the absence of chemical and biologic activity at the storage temperature but we only tested out to 6 months to prove the concept. According to the US Food and Drug Administration (FDA) guideline for donor eligibility, tissue donors for clinical transplantation are required to be screened and identified for the potential of disease transmission^6,8,41^. The testing is performed on every tissue to confirm the safety of the tissue donated for clinical transplantation. However, these tests take ~14 days to complete, which, given the decline in chondrocyte function and viability after this time, makes the time frame for scheduling operations very short for surgeons in practice. In our study, we validated a stepwise vitrification approach using porcine articular cartilage, and further translated the method to human articular cartilage, providing a promising outcome that meets the requirement of 70% chondrocyte viability for clinical transplantation: our vitrification approach yields a high (>80%) normalized chondrocyte viability in human particulated articular cartilage, a critical result for successful clinical implementation and use.

Particulated articular cartilage grafting is an accepted method of clinical chondral repair. Performed as a single-stage procedure, small particulated articular cartilage allograft (from a deceased donor) is used to fill cartilage defects using fibrin fixation, such that a new articulating surface is developed in joints such as knees and ankles^4,42^. Currently, the most used particulated cartilage grafts are provided by Zimmer, branded as DeNovo^®^ NT (natural tissue) graft. A follow-up of 25 patients aged 37 ± 11 years reported significant improvement in clinical outcomes as well as histologically indicated repair of hyaline-like cartilage after 24 months^43^. Another mid-term follow-up study showed a progressive improvement in sequential MRIs with 70% achieving complete filling of knee cartilage defects in 26 patients after 4.4 years^44^. Although many cases using particulated articular cartilage have been performed on patients with full-thickness chondral defects, this treatment is limited by the availability of viable articular cartilage. In this study, based on mathematical modeling, we provide effective protocols to preserve particulated articular cartilage (~1 mm^3^ in size) with high viability of chondrocytes in porcine and human articular cartilage and validate their functionalities experimentally. Chondrocytes in porcine particulated articular cartilage migrated out of the cellular matrix and proliferated in vitro as shown in Fig. 4a; this evidence is consistent with the experiments reported by Tompkins et al.^2^ for human particulated articular cartilage, an important property for cartilage repair. In our study, we further isolated the chondrocytes from vitrified articular cartilage to assess their proliferation and synthesis capacities: our results showed that the chondrocytes isolated from vitrified articular cartilage of both porcine and human species maintain a similar capacity when compared to their respective fresh control chondrocytes that did not undergo vitrification and rewarming. With our current findings, vitrified particulated articular cartilage can be a potential source of tissue for use in clinical cartilage repair. Our results encourage an in vivo animal transplantation study to validate the effectiveness of cartilage repair using these vitrified particulated articular cartilage, and to guide the clinical translation of our approach for repairing human articular cartilage defects.

The chondrocyte viability of human particulated articular cartilage declined by ~15–20% after vitrification when compared to the fresh control group. In contrast, the porcine samples’ viability declined only by ~5–10%. This difference may be attributed to the quality of the donated human tissue (although we have healthy articular cartilage with a positive control viability > 90%), one factor that may affect cartilage quality being the age of donor. Articular cartilage becomes mature in humans at the age of 20 years^45,46^, characterized by a significant deposition of GAGs in the extracellular matrix. As age increases to 50–60 years, proteoglycans start to degenerate^47^ and chondrocyte density decreases in comparison to articular cartilage in children aged younger than 13 years^2^. Over time, aged chondrocytes undergo slow apoptosis that worsens with more frequent and more intense daily activity accumulated over several decades, with a significant loss of water content in the cartilage matrix^48^. These changes to the cartilage increase the sensitivity and survival of chondrocytes when exposed to extracellular stimuli compared to that of chondrocytes in juvenile articular cartilage. In this study, we collected human articular cartilage from three donors (two male and one female, age: 48, 50, 59 years), ages at which cartilage quality would be expected to have declined. Conversely, the pigs are sexually mature but would be considered the equivalent to teenagers or young adults relative to humans. Second, the length of time between tissue harvesting and laboratory use can be detrimental to cell survival. In this study, the human articular cartilage from the three donors was harvested ~24 h after donor death and kept in the tissue bank before same day delivery to the lab. Our previous research showed that porcine articular cartilage stored at 4 °C for 24 h after slaughter and harvest had a lower tolerance to the chemical toxicity of a high concentration of cryoprotectants^49^, when compared to those slaughtered and delivered to the lab within 6 h, which could be due to the reactive oxidative species produced during the cryoprotectant loading procedure^50^. Therefore, it is likely that the lower chondrocyte viability of human tissues compared to porcine tissues in this study is due to both donor age and processing time of human articular cartilage. To improve chondrocyte viability in the clinical setting, juvenile or young adult articular cartilage could be vitrified, and a processing protocol in the tissue bank to guide the vitrification of human articular cartilage needs to be established in a future investigation.

## Methods

### Protocol calculation

To design our loading protocols, we calculated the spatial and temporal distribution of cryoprotectant concentration, solution freezing point, and vitrifiability, bringing together the work of Abazari et al.^28,37^ on diffusion coefficients; the work of Elliott et al.^26,27,29,30^ on the multisolute osmotic virial equation for predicting freezing point; and the work of Weiss et al.^25^ on solution vitrifiability. We first presented this three-part mathematical approach in a previous paper where we proposed an optimal protocol for preserving articular cartilage dowels^23^ that was shown to yield high cell viability when implemented experimentally^32^. Herein, we use our three-part model to develop protocols for articular cartilage cubes.

The first step of developing a vitrification protocol is to choose the cryoprotectant types and concentrations. Protocol E–D–P is based on our previous work on articular cartilage dowels^23^, and as we did there, here we select EG, DMSO, and PG to load at progressively lower temperatures. In the first loading step at a temperature of 0 °C, we select EG and DMSO because these permeate the quickest and depress the freezing point quickly for the subsequent loading step to take place. In the second loading step, PG is added. In both steps, the concentrations of EG and DMSO are 3 M (the same concentration as we used previously for cartilage dowels^23^ based on experimental evidence that 3 M solutions of these cryoprotectants are associated with low toxicity^35^), while the concentration of PG is 2 M (a concentration selected to permit the attainment of a vitrifiable concentration at the end of the loading protocol within a reasonable time period).

Protocol E–D is designed to avoid the use of PG, which has been identified as more toxic than EG and DMSO. For this protocol, we select a loading solution of 3 M EG in the first loading step at a temperature of 0 °C. In the second loading step, we select a solution with DMSO and EG both at 4 M (a concentration selected to expedite the diffusion into the cartilage for the protocol to take a similar amount of time as Protocol E–D–P).

Given the above defined concentrations for the loading solutions of each protocol, the only remaining variables to be defined are the times of each loading step and the temperature of the second loading step. From the practical perspective, we note that a single loading step cannot be less than 10 min long because of the time taken for the experimental procedure. Therefore, we vary the time of each loading step between 10 and 40 min in intervals of 5 min. For the initial iteration of protocol development, we set the temperature of the second step to be 0 °C, and we calculate the expected spatial and temporal distributions of cryoprotectant concentration, solution freezing point, and solution vitrifiability for each combination of loading step times. Of all the possible protocols, we select the shortest protocol that predicts a vitrifiability score at all spatial points in the cartilage to exceed the minimum vitrifiability score. Based on the predicted maximum freezing point at the end of this protocol’s first step, we then choose a temperature for the second loading step that is greater than this freezing point. Finally, we confirm that this new temperature also ensures that all spatial points in the cartilage remain vitrifiable.

### Cryoprotectant concentration

Fick’s law of diffusion is used in one dimension to calculate the spatial and temporal evolution of cryoprotectant permeation

1 $$\frac{{\partial C}}{{\partial t}} = D\frac{{\partial ^2C}}{{\partial x^2}}$$

where *C* is cryoprotectant concentration (mol/L), *t* is time (s), *D* is the diffusion coefficient (m^2^/s), and *x* is position in the cartilage. The diffusion coefficient is calculated as a function of absolute temperature (*T*) using the Arrhenius expression

2 $$D = A\exp \left( { - \frac{{E_a}}{{RT}}} \right)$$

where *A* is the pre-exponential factor (m^2^/s), *E*_*a*_ is the activation energy (kcal/mol), as listed in Table 3, for each cryoprotectant, and *R* is the ideal gas constant.

**Table 3:** Coefficients for use in calculating the diffusion coefficient of each cryoprotectant in porcine cartilage (Eq. 2)^28^.

	*A* (m^2^/s)	*E*_*a*_ (kcal/mol)
DMSO	2.9895 × 10^−7^	3.9 ± 1.6
PG	1.6971 × 10^−5^	6.63 ± 0.04
EG	1.833 × 10^−7^	3.8 ± 0.7

Experimentally, condyles are minced into cubes with side lengths of 1 mm. Based on this geometry, we consider a one-dimensional line from the center of one cube face to the center of the cube (length of 0.5 mm) for our calculations, as illustrated in Fig. 1a. We select this dimension for our diffusion calculations because 0.5 mm is the shortest distance from the cartilage–solution boundary to the center of the cube. Using this geometry, the calculated concentration at the center is a lower bound on the expected concentration, given that diffusion also occurs from each side of the exposed cube.

The following boundary and initial conditions are used to solve Eq. (1) for calculating the spatial and temporal distribution of cryoprotectant concentration during loading. First, the concentration of each cryoprotectant at the outer surface of the cube is equal to the concentration in the solution

3 $$C\left( {x = 0.5{\mathrm{mm}},t} \right) = C_{{\mathrm{solution}}}$$

At the center of the cube, there is no flow of cryoprotectant

4 $$\frac{{\partial C}}{{\partial x}}\left( {x = 0\,{\mathrm{mm}},t} \right) = 0$$

Finally, the initial condition at the beginning of the first loading step is

5 $$C\left( {0 \,<\, x \,<\, 0.5\,{\mathrm{mm}},t = 0} \right) = 0$$

The concentration profile of each cryoprotectant at the end of the first loading step is used as the initial condition for the second loading step.

### Vitrifiability

Given the distribution of cryoprotectant concentration calculated using Eq. (1), the corresponding spatial distribution of vitrifiability can be determined for the volume and cooling rate expected during the vitrification of cartilage cubes. For our predictions, we use the statistical model of vitrifiability developed by Weiss et al.^25^, where 5 mL solutions containing between 6 and 9 M of cryoprotectants were placed in 10 mL polypropylene tubes and plunged into liquid nitrogen (a ~60 K/min cooling rate^25,31^) for 30 min, and then immersed in a 37 °C water bath until they liquified completely, for which an ordinal score was assigned based on visual inspection (on a scale from 0 to 4 outlined in Table 4). The following statistical model was developed with proportional odds logistic regression based on the experimental scores of ordinal vitrifiability for 164 cryoprotectant solutions^25^

6 $$\mathop {\sum }\limits_{i = 1}^p \left[ {\beta _iC_i + \mathop {\sum }\limits_{j = 1}^i \beta _{ij}C_iC_j} \right] \ge \left| {\alpha _n} \right|$$

where *β*_*i*_ and *β*_*ij*_ are single and interaction (including self-interaction) coefficients for each cryoprotectant *i* and cryoprotectant pair *ij*, respectively, as listed in Table 5^25^, and *C*_*i*_ is the molar concentration (mol/L). If the summation calculated over the *p* cryoprotectants exceeds a threshold value of *α*_*n*_, the solution will reach a vitrifiability score of at least *n* (Table 4). The coefficients listed in Table 5 are only valid for predicting vitrifiability under the same or more favorable conditions as the experiments in Weiss et al.^25^, i.e., for 5 mL solutions in 10 mL polypropylene tubes cooled at ~60 K/min and thawed in a 37 °C water bath. Herein, cartilage cubes are placed into a 1.8 mL Cryovial tube for vitrification and warming, which is a smaller volume than that used by Weiss et al.^25^. A smaller volume increases the rate of cooling and warming and improves the vitrifiability of the cryoprotectant solution within the cartilage matrix. Thus, any protocol that satisfies the vitrifiability criterion determined by Weiss et al.^25^ is also expected to be vitrifiable under the experimental conditions used herein.

**Table 4:** Ordinal scores for the ordinal model of vitrifiability^25^.

Ordinal score	Description
0	No vitrification
1	Complete devitrification
2	Partial devitrification
3	Devitrification at edges
4	No devitrification

**Table 5:** Numerical thresholds (*α*) and coefficients (*β*) for the ordinal model of vitrifiability^25^.

Parameter	Estimate
*α*_1_	167.6 ± 29.2
*α*_2_	184.0 ± 31.5
*α*_3_	186.8 ± 31.8
*α*_4_	190.5 ± 32.0
*β*_PG_	57.1 ± 10.6
*β*_EG_	39.9 ± 8.0
*β*_*DMSO*_	36.4 ± 6.7
*β*_PG_EG_	−5.9 ± 1.3
*β*_DMSO_PG_	−5.9 ± 1.2
*β*_PG_PG_	−4.1 ± 0.9
*β*_DMSO_EG_	−3.7 ± 0.9
*β*_EG_EG_	−2.3 ± 0.6
*β*_DMSO_DMSO_	−1.4 ± 0.4

### Freezing point

The distribution of freezing point as a function of position in the cartilage is calculated using^26,27,30^

7 $$T_{{\mathrm{FP}}}^0 - T_{{\mathrm{FP}}} = \frac{{\left[ {W_1/\left( {\overline {s_1^{0{\mathrm{L}}}} - \overline {s_1^{0{\mathrm{S}}}} } \right)} \right]RT_{{\mathrm{FP}}}^0\pi }}{{1 + \left[ {W_1/\left( {\overline {s_1^{0{\mathrm{L}}}} - \overline {s_1^{0{\mathrm{S}}}} } \right)} \right]R\pi }}$$

where $T_{{\mathrm{FP}}}^0$ is the freezing point of pure water (273.15 K), *T*_FP_ is the freezing point of the cryoprotectant mixture, *π* is the osmolality (osmol/kg solvent), *W*_1_ is the molar mass of pure water, and $\overline {s_1^{0{\mathrm{L}}}} - \overline {s_1^{0{\mathrm{S}}}} = 22.00$ J/mol K is the change in molar entropy between pure liquid and pure solid water at $T_{{\mathrm{FP}}}^0$.

The multisolute osmotic virial equation^26,27,29,30^ is used to calculate solution osmolality with the fitting coefficients determined by Zielinski et al.^26^. Only the second-order virial coefficients (*B*) are needed to accurately describe cryoprotectant solutions containing DMSO, PG, and EG with an isotonic amount of NaCl

8 $$\pi = \mathop {\sum }\limits_{i = 2}^r k_im_i + \mathop {\sum }\limits_{i = 2}^r \mathop {\sum }\limits_{j = 2}^r \frac{{B_i + B_j}}{2}k_im_ik_jm_j$$

where *k* is the dissociation constant and *m* is the molality, which is calculated with

9 $$m_i = \frac{{\left( {1000\frac{{\mathrm{L}}}{{{\mathrm{m}}^3}}} \right)C_i}}{{\rho _1\left[ {1 - \mathop {\sum }\nolimits_{i = 2}^{r - 1} C_iV_{m,i}} \right]}}$$

where *ρ*_1_ is the density of pure water (998 kg/m^3^ at 22 °C^51^) and *V*_*m*_ is the molar volume (L/mol) at 22 °C^52^. Dissociation constants, osmotic virial coefficients, and molar volumes are listed in Table 6 for each cryoprotectant. The volume of mixing and the volumes of NaCl (and other minute additives) are assumed negligible in Eq. (9).

**Table 6:** Dissociation constants and virial coefficients for Eq. (8) ^26^ and the molar volume^52^ of each cryoprotectant for Eq. (9).

	*k*	*B* (molal^−1^)	*V*_*m*_ (L/mol)
DMSO	1	0.108 ± 0.005	0.0709
PG	1	0.039 ± 0.001	0.0735
EG	1	0.020 ± 0.001	0.0559
NaCl	1.678	0.044 ± 0.002	–

### Porcine articular cartilage cube preparation

Hind legs with joints (*n* = 23 porcine hind legs) from sexually mature pigs aged over 54 weeks were obtained from a meat-processing plant slaughter house in Wetaskiwin, AB, Canada. No animals were specifically euthanized for this research. The Research Ethics Office at the University of Alberta provided ethical approval for the experimental use of animal tissues and cells. Porcine joints were harvested and immersed in phosphate buffered saline (PBS) immediately, then transported to the research laboratory within 4 h. After joint dissection, articular cartilage was shaved from the condyles, and minced into cubes ~1 mm^3^ in size using a sterile scalpel blade and cleaned with 50 mL sterile PBS (Ca^2+^/Mg^2+^ free) plus antibiotics [100 units/mL penicillin, 100 µg/mL streptomycin, and 0.25 µg/mL amphotericin B (Gibco)] for 15 min under a biological safety cabinet (NuAire, MN, USA).

### Human articular cartilage cube preparation

Healthy knee joints (*n* = 3 donors) from deceased donors aged 48, 50, and 59 years (mean ± SD: 52.3 ± 5.8 years) were obtained from the Comprehensive Tissue Center in Edmonton, AB, Canada, with consent from patients’ families to use donated cartilage for research. Human research ethics approval was obtained from the University of Alberta Research Ethics Office. After tissue harvesting, knee joints were stored in 500 mL X-VIVO 10 (Lonza, California, USA, a serum-free medium (SFM) that has been approved for clinical use) and transported to the research laboratory within 24 h. Healthy articular cartilage was shaved from the condyles, minced into cubes ~1 mm^3^ in size using a sterile scalpel blade, and immediately immersed in PBS, then cleaned with 50 mL sterile PBS (Ca^2+^/Mg^2+^ free) plus antibiotics [100 units/mL penicillin, 100 µg/mL streptomycin (Gibco)] for 15 min under a biological safety cabinet.

### Cryoprotectant cocktail solution preparation and stepwise cryoprotectant loading protocol

Multicryoprotectant cocktail solutions were made from three cryoprotectants: EG (Fisher), DMSO (Fisher), and PG (Fisher). Fresh cryoprotectant cocktail solutions were prepared in 50 mL final volumes with Dulbecco’s Modified Eagle Medium F12 (DMEM) (Gibco) for porcine cartilage or with X-VIVO 10 SFM for human cartilage on the same day of the experiment, using the following concentrations of cryoprotectants (M = molar): Protocol E–D–P: solution one [3 M EG + 3 M DMSO] and solution two [3 M EG + 3 M DMSO + 2 M PG] and Protocol E–D: solution one [3 M EG] and solution two [4 M EG + 4 M DMSO]. After weighing, cartilage cubes were transferred into the prepared 50 mL cryoprotectant cocktail solutions for cryoprotectant permeation at specific temperatures and times: Protocol E–D–P: solution one at 0 °C for 10 min, followed by solution two at −10 °C for 20 min and Protocol E–D: solution one at 0 °C for 20 min, followed by solution two at −5 °C for 15 min. After the cryoprotectant loading into the cartilage, the particulated cartilage cubes were quickly removed from the Falcon tubes with a mesh strainer and transferred into a sterile 1.8 mL Cryovial tube using a chemical spoon. After closing the vial lid, the Cryovial tube was placed onto a Cryovial cane and quickly plunged into liquid nitrogen for vitrification.

### Cryoprotectant removal from articular cartilage cubes

The Cryovial tube containing vitrified cartilage cubes was quickly removed from the liquid nitrogen and warmed in a 37 °C water bath until the surrounding glass was melted (~0.5 min). The cartilage cubes were extracted with a sterile spatula and washed three times in 25 mL DMEM (for porcine cartilage) or 25 mL X-VIVO 10 SFM (for human cartilage) for 30 min each wash to remove the permeated cryoprotectants from the cartilage cubes.

### Temperature profile of vitrification and rewarming processes

To compare the cooling and warming temperature profiles of Protocol E–D–P and Protocol E–D, a dual thermometer with a thermocouple detector was used to measure the temperatures of 0.5 g of articular cartilage cubes after cryoprotectant permeation within the 1.8 mL Cryovial (*n* = 5 replicates per group) as a function of time as they were cooled from the step two temperature (−10 or −5 °C) to −196 °C in liquid nitrogen for 10 min, then rewarmed to 37 °C in a water bath for 30 s and transferred to a 4 °C wash media. The temperatures of articular cartilage at the following time points (for cooling, at 0, 10, 20, 30, 40, 50, 60, 90, 120, and 180 s; for rewarming, at 600, 610, 620, 630, 660, 720, 780, 840, and 900 s) were recorded and plotted to show the thermal history regarding the cooling and warming of each protocol.

### Chondrocyte viability by cell membrane integrity stain

Chondrocyte viability was assessed by a cell membrane integrity stain [6.25 µM Syto 13 and 9.0 µM propidium iodide mixed in PBS] using a membrane-permeant nucleic acid stain (Syto 13; Molecular Probes) which fluoresced green, and a membrane-impermeant stain (propidium iodide; Sigma) that penetrates only into cells with disrupted cell membranes fluorescing red. After incubation of cartilage cubes in the dyes for 20 min, cartilage cubes were rinsed in PBS (Ca^2+^/Mg^2+^ free) and imaged using a laser scanning confocal fluorescent microscope (model: TCS SP5; Leica). The filters used to image all the cartilage cubes in this study had the following spectra peak maxima wavelengths: excitation/emission: 488 nm/503 nm and 535 nm/617 nm. Three replicate cartilage cubes from one Cryovial were imaged at each time point. Cartilage cubes were imaged at three time points, *t*_1_ = positive control before cryoprotectant loading (fresh cartilage cubes after mincing), *t*_2_ = after vitrification in LN_2_ (<24 h, day 0) and tissue warming followed by cryoprotectant removal in medium, and *t*_3_ = after vitrification for 180 days (day 180) and tissue warming followed by cryoprotectant removal in medium. A positive control (fresh cartilage cubes after mincing) and a negative control group (freeze/thaw in liquid nitrogen (LN_2_) without cryoprotectants) from the same condyle were assessed. A minimum 80% absolute chondrocyte viability in the positive (fresh) controls before cryoprotectant exposure (chondrocyte viability at *t*_1_) was used to screen out unhealthy cartilage donors.

### Chondrocyte metabolic activity by alamarBlue

Chondrocyte metabolic activity was assessed by an alamarBlue assay (Invitrogen, Burlington). Rewarmed articular cartilage cubes (~0.2 g wet weight) after cryoprotectant removal were washed in 5 mL sterile PBS (Ca^2+^/Mg^2+^ free) plus antibiotics for 15 min in a biological safety cabinet. Cartilage cubes were incubated with the alamarBlue assay solution [5 mL X-VIVO 10 medium supplemented with 0.1 mM ascorbic acid, 10 nM dexamesasone, 10 ng/mL transforming growth factor (TGF) beta 1, and mixed with 500 µL alamarBlue] at 37 °C for 48 h. Images of the fluorescence color change of alamarBlue assay solutions of the culture plates were taken at 0, 24, and 48 h using a digital camera (Canon PowerShot ELPH 180). The average of two replicate readings of the blank samples (alamarBlue assay solution without cartilage sample) was subtracted from the average of the experimental samples to yield a value in relative fluorescent units (RFU) divided by gram weight. The RFU were determined by the CytoFluor II software with emission wavelengths of 580/50 nm, excitation wavelengths of 485/20 nm, and a fluorescent intensity gain set to 45.

### Articular cartilage digestion for chondrocyte isolation

After cryoprotectant removal, 0.2 g of porcine cartilage cubes or 0.5 g of human cartilage cubes were weighed and cleaned with 5 mL sterile PBS (Ca^2+^/Mg^2+^ free) plus antibiotics [100 units/mL penicillin, 100 µg/mL streptomycin (Gibco)] for 15 min under a biological safety cabinet. The cartilage cubes were then transferred to an empty 50 mL Falcon tube and 5 mL of 0.15% collagenase solution was added under sterile conditions [for 10.5 mL of collagenase solution, prepare: 10 mL DMEM supplemented with antibiotics (PS), 15 mg of 300 units type II collagenase (filtered, Worthington), and 0.5 mL fetal bovine serum (FBS)]. The Falcon tubes containing cartilage cubes were placed in an orbital shaker (250 rpm) at 37 °C for cartilage digestion for 22 h. Once the cartilage digestion was finished, a sterile 100 µm cell strainer was used to filter the digested chondrocytes. The collagenase was neutralized by adding 10 mL of DMEM supplemented with 10% FBS. The chondrocytes were collected by centrifugation for 10 min at 433 × *g* at 22 °C, followed by two washes in 10 mL sterile PBS (Ca^2+^/Mg^2+^ free), and then resuspended in 12 mL of DMEM complete for chondrocyte recovery.

### Chondrocyte recovery and chondrocyte collection

After chondrocyte recovery in an appropriate tissue culture flask (BD, Falcon) in a humidified incubator with 20% O_2_ and 5% CO_2_ at 37 °C for 72 h, the chondrocyte monolayer was washed with 5 mL sterile PBS (Ca^2+^/Mg^2+^ free) twice. 2 mL 1 × 0.02% trypsin-EDTA solution (Gibco) was added to the tissue culture flask to disassociate chondrocytes for 5 min at 37 °C, and then neutralized with 5 mL of DMEM complete supplemented with 10% FBS. Chondrocytes were collected for cell counting via centrifugation for 10 min at 433 × *g* at 22 °C.

### Chondrocyte counting by trypan blue

After chondrocytes were resuspended in DMEM complete media, 15 µL cell suspension and 15 µL trypan blue were mixed by pipetting. Ten microliters of this mixture was gently placed in a hematocytometer using a pipette for chondrocyte counting, and the cell count was determined by adding the counted cells in four equally sized areas, dividing by 4, and then multiplying by a dilution factor of 10,000 and by the total volume of chondrocyte suspension solution. Trypan blue is a vital stain used to selectively color dead cells with a blue color, and live cells with intact cell membranes remain unstained. Since live chondrocytes are excluded from staining, this staining method can be used as a dye exclusion method to identify the number of living chondrocytes.

### Scratch wound healing assay and chondrocyte migration quantification

After chondrocyte recovery for 72 h, chondrocytes were counted with trypan blue and seeded onto a 24-well tissue culture plate (Aaka Scientific Inc.) with a density of 10^5^ per well and cultured in a humidified incubator with 20% O_2_ and 5% CO_2_ at 37 °C for 168 h. Chondrocytes were grown until they reached over 90% confluence as a monolayer in the culture plate in 2 mL DMEM complete supplemented with 10% FBS with the medium changed twice a week. For the scratch wound healing assay, a sterile 200 µL pipette tip was used to slowly scratch the confluent monolayer (90% or higher) from left to right across the center of the well and introduce a 1 mm wide empty gap in the wells^53^. The wells were refilled with 2 mL fresh DMEM complete and images of the well were taken at 0, 24, and 48 h to monitor the migration of chondrocytes. Image J software was used to calculate chondrocyte migration percentage every 24 h. Chondrocyte migration was normalized to the initial empty gap width at 0 h and plotted to show the chondrocyte migration speed based on the 24 and 48 h time points.

### Chondrocyte aggregate by pellet culture for 21 days

After isolated chondrocytes were plated for 72 h for cell recovery, chondrocytes were washed with sterile PBS (Ca^2+^/Mg^2+^ free) twice. Then, chondrocytes were trypsinized for 5 min at 37 °C and centrifuged at 433 × *g* for 5 min to collect chondrocytes for making pellets following the procedure below^54^. After a cell wash and cell counting with trypan blue, 5 × 10^5^ chondrocytes were resuspended in 500 µL defined chondrogenic SFM [high glucose DMEM, HEPES (10 mM), human serum albumin (125 mg/mL), ascorbic acid 2-phosphate (365 lg/mL), dexamethasone (100 nM), and L-proline (40 lg/mL) (Sigma-Aldrich), ITS + 1 premix (5 µL, 100x) (Corning, Discovery Labware, Inc.), 100 units/mL penicillin, 100 µg/mL streptomycin, TGF-b3 10 ng/mL; ProSpec, NJ, USA] in a 1.5 mL sterile conical microtube (Bio Basic Inc, Ontario, Canada). Then, chondrocytes were centrifuged at 433 × *g* and 22 °C for 5 min to form a pellet at the bottom of the microtube using an Allegra X-22R centrifuge (Beckman Coulter, US). The pellets were cultured in the SFM under 3% O_2_ and 5% CO_2_ at 37 °C in a humidified incubator for 21 days, with SFM changes twice a week.

### Wet weight and histology of pellets

After a 21-day culture, pellets were rinsed with sterile PBS (Ca^2+^/Mg^2+^ free) and wet weights were measured using an electric balance (Mettler Toledo, Switzerland). Pellets were imaged with a Zeiss camera (AxioCam ERc 5s) for gross morphology and fixed with 10% formalin for 24 h before paraffin embedding. A microtome (Leica) was used to prepare pellet sections with thicknesses of 5 µm, followed by section drying at 37 °C overnight in a dry incubator. Pellet sections were then processed with Safranin O staining to quantify and identify proteoglycan content in the pellets. Stained sections were imaged with a Nikon digital camera (model: DS-Fi2) equipped on a Nikon inverted microscope (model: ECLIPSE Ti-5): exposure time for 100× magnification = 8 ms; exposure time for 200× magnification = 40 ms; gain = 0.

### GAG/DNA measurement

GAG content of pellets was quantified by a dimethylmethylene blue (DMMB) assay. Pellets were weighed and rinsed with PBS and stored in a −80 °C freezer before use. After warming, pellets were digested in 250 µL of 1 mg/mL proteinase K overnight at 56 °C using a dry block heater (Thermo Fisher Scientific). PBE/cysteine buffer (100 mM Na_2_HPO_4_, 10 mM Na_2_EDTA, pH = 6.5, 1.75 mg/mL cysteine, Sigma) and ~0–100 µg/mL chondroitin sulfate A sodium salt (CS, Sigma-Aldrich) were used as controls for a standard curve. The standard curve was prepared in eight Eppendorf tubes with a total volume of 100 µL and an increasing concentration of CS. After protein digestion, a 5 µL digested sample was pipetted into an ultraclear 96-well plate in triplicate (NUNC, Thermo Fisher Scientific). The digested sample in each well was mixed with 5 µL PBE/cysteine buffer and diluted by 1:50 in concentration by adding 250 µL DMMB dye (Sigma-Aldrich). Each plate was read at 525 nm and data were normalized to the blank reading of H_2_O (260 µL, without DMMB) and the CS standard controls. DNA content was quantified by using the CyQUANT^TM^ proliferation assay kit for cells in culture (Invitrogen, ON, Canada). After cartilage digestion with 1 mg/mL proteinase K, a 5-µL sample was pipetted into a 96-well plate in triplicate. In each well, 195 µL working buffer was added to make the total volume equal to 200 µL. DNA solutions and working buffer were prepared using an assay kit^54^. For 20 mL working buffer, 50 µL CyQUANT^®^ GR dye and 1 mL cell-lysis buffer were mixed with 19 mL Milli-Q water. The spectra peak maxima for excitation of 450/50 nm and emission of 530/25 nm were used to read the plates, and the supplied λDNA of bacteriophage was used as standard reference.

### Statistical analysis

The numerical data are presented as means ± SD. Based on the Mauchly’s test of sphericity or the Levene’s test of equality, the analysis of variance (ANOVA) with post hoc test (Tukey’s multiple comparison) was performed on the experimental groups, otherwise, the nonparametric test (Kruskal–Wallis with pairwise comparison) was performed to compare experimental variables in multiple groups. Sample size and the *p* values are reported in the figure legends. All data were analyzed using SPSS 20.0 software for statistical significance and figures were plotted using GraphPad Prism 8 software.

### Reporting summary

Further information on research design is available in the Nature Research Reporting Summary linked to this article.

## Supplementary information

Supplementary material

Reporting Summary Checklist

## Data Availability

All experimental data are provided in the “Results” or Supplementary Materials sections. The chemicals and tools required for the experiments are indicated in the “Methods” section.

## Code availability

Calculations were made with MATLAB (version R2019a); all pertinent details of the calculations are listed in the “Methods” section.

## References

[1] Hunziker EB (2002). Articular cartilage repair: basic science and clinical progress. a review of the current status and prospects. Osteoarthr. Cartil..

[2] Tompkins M, Adkisson HD, Bonner KF (2013). DeNovo NT allograft. Oper. Tech. Sports Med..

[3] McCormick F, Yanke A, Provencher MT, Cole BJ (2008). Minced articular cartilage—basic science, surgical technique, and clinical application. Sports Med. Arthrosc..

[4] Riboh JC, Cole BJ, Farr J (2015). Particulated articular cartilage for symptomatic chondral defects of the knee. Curr. Rev. Musculoskelet. Med..

[5] Riff, A. J., Davey, A. & Cole, B. J. Emerging technologies in cartilage restoration. in *Joint Preservation of the Knee: a Clinical Casebook*, 295–319 (Springer International Publishing, 2019). 10.1007/978-3-030-01491-9_18.

[6] Görtz S, Bugbee WD (2006). Fresh osteochondral allografts: graft processing and clinical applications. J. Knee Surg..

[7] Williams RJ, Dreese JC, Chen CT (2004). Chondrocyte survival and material properties of hypothermically stored cartilage: an evaluation of tissue used for osteochondral allograft transplantation. Am. J. Sports Med..

[8] Goodfriend B, Essilfie AA, Jones IA, Thomas Vangsness C (2019). Fresh osteochondral grafting in the United States: the current status of tissue banking processing. Cell Tissue Bank..

[9] Abazari A, Jomha NM, Elliott JAW, McGann LE (2013). Cryopreservation of articular cartilage. Cryobiology.

[10] Muldrew K, McGann LE (1994). The osmotic rupture hypothesis of intracellular freezing injury. Biophys. J..

[11] Muldrew K (2000). Cryobiology of articular cartilage: ice morphology and recovery of chondrocytes. Cryobiology.

[12] Fahy GM, MacFarlane DR, Angell CA, Meryman HT (1984). Vitrification as an approach to cryopreservation. Cryobiology.

[13] Jomha NM (2012). Vitrification of intact human articular cartilage. Biomaterials.

[14] Pegg DE, Wang L, Vaughan D (2006). Cryopreservation of articular cartilage. Part 3: the liquidus-tracking method. Cryobiology.

[15] Song YC, Lightfoot FG, Chen Z, Taylor MJ, Brockbank KGM (2004). Vitreous preservation of rabbit articular cartilage. Cell Preserv. Technol..

[16] Brockbank KGM, Chen ZZ, Song YC (2010). Vitrification of porcine articular cartilage. Cryobiology.

[17] Abazari A, Elliott JAW, Law GK, McGann LE, Jomha NM (2009). A biomechanical triphasic approach to the transport of nondilute solutions in articular cartilage. Biophys. J..

[18] Abazari A, Thompson RB, Elliott JAW, McGann LE (2012). Transport phenomena in articular cartilage cryopreservation as predicted by the modified triphasic model and the effect of natural inhomogeneities. Biophys. J..

[19] Abazari A, Elliott JAW, McGann LE, Thompson RB (2012). MR spectroscopy measurement of the diffusion of dimethyl sulfoxide in articular cartilage and comparison to theoretical predictions. Osteoarthr. Cartil..

[20] Lawson A, Mukherjee I, Sambanis A (2012). Mathematical modeling of cryoprotectant addition and removal for the cryopreservation of engineered or natural tissues. Cryobiology.

[21] Mukherjee IN, Li Y, Song YC, Long RC, Sambanis A (2008). Cryoprotectant transport through articular cartilage for long-term storage: experimental and modeling studies. Osteoarthr. Cartil..

[22] Shardt N (2016). Cryoprotectant kinetic analysis of a human articular cartilage vitrification protocol. Cryobiology.

[23] Shardt N (2020). Using engineering models to shorten cryoprotectant loading time for the vitrification of articular cartilage. Cryobiology.

[24] Benson JD, Kearsley AJ, Higgins AZ (2012). Mathematical optimization of procedures for cryoprotectant equilibration using a toxicity cost function. Cryobiology.

[25] Weiss ADH (2010). Statistical prediction of the vitrifiability and glass stability of multi-component cryoprotective agent solutions. Cryobiology.

[26] Zielinski MW, McGann LE, Nychka JA, Elliott JAW (2014). Comparison of non-ideal solution theories for multi-solute solutions in cryobiology and tabulation of required coefficients. Cryobiology.

[27] Elliott JAW, Prickett RC, Elmoazzen HY, Porter KR, McGann LE (2007). A multisolute osmotic virial equation for solutions of interest in biology. J. Phys. Chem. B.

[28] Abazari A, Jomha NM, Law GK, Elliott JAW, McGann LE (2009). Erratum to ‘Permeation of several cryoprotectants in porcine articular cartilage’ [Cryobiology 58 (2009) 110-114]. Cryobiology.

[29] Prickett RC, Elliott JAW, McGann LE (2011). Application of the multisolute osmotic virial equation to solutions containing electrolytes. J. Phys. Chem. B.

[30] Prickett RC, Elliott JAW, McGann LE (2010). Application of the osmotic virial equation in cryobiology. Cryobiology.

[31] Jomha NM, Anoop PC, McGann LE (2004). Intramatrix events during cryopreservation of porcine articular cartilage using rapid cooling. J. Orthop. Res..

[32] Wu K (2020). Comparison of three multi-cryoprotectant loading protocols for vitrification of porcine articular cartilage. Cryobiology.

[33] Macfadyen MA (2019). The commercial pig as a model of spontaneously-occurring osteoarthritis. BMC Musculoskelet. Disord..

[34] Almansoori KA (2012). Cryoprotective agent toxicity interactions in human articular chondrocytes. Cryobiology.

[35] Jomha NM (2010). Cryoprotectant agent toxicity in porcine articular chondrocytes. Cryobiology.

[36] Fahmy MD (2014). Dose-injury relationships for cryoprotective agent injury to human chondrocytes. Cryobiology.

[37] Jomha NM (2009). Permeation of several cryoprotectant agents into porcine articular cartilage. Cryobiology.

[38] Bugbee WD, Pallante-Kichura AL, Görtz S, Amiel D, Sah R (2016). Osteochondral allograft transplantation in cartilage repair: graft storage paradigm, translational models, and clinical applications. J. Orthop. Res..

[39] Zachari MA (2014). Evaluation of the alamarblue assay for adherent cell irradiation experiments. Dose-Response.

[40] Shum D (2008). A high density assay format for the detection of novel cytotoxic agents in large chemical libraries. J. Enzym. Inhib. Med. Chem..

[41] Levine DW, Mondano L, Halpin M (2008). FDA regulatory pathways for knee cartilage repair products. Sports Med. Arthrosc..

[42] Hatic SO, Berlet GC (2010). Particulated juvenile articular cartilage graft (DeNovo NT Graft) for treatment of osteochondral lesions of the talus. Foot Ankle Spec..

[43] Farr J, Tabet SK, Margerrison E, Cole BJ (2014). Clinical, radiographic, and histological outcomes after cartilage repair with particulated juvenile articular cartilage. Am. J. Sports Med..

[44] Patterson D (2017). Mid-term results of particulated juvenile articular cartilage allograft transplantation to the knee. Arthrosc. J. Arthrosc. Relat. Surg..

[45] Temple MM (2007). Age- and site-associated biomechanical weakening of human articular cartilage of the femoral condyle. Osteoarthr. Cartil..

[46] Kempson GE (1982). Relationship between the tensile properties of articular cartilage from the human knee and age. Ann. Rheum. Dis..

[47] Wells T (2003). Age-related changes in the composition, the molecular stoichiometry and the stability of proteoglycan aggregates extracted from human articular cartilage. Biochem. J..

[48] Loeser RF (2009). Aging and osteoarthritis: the role of chondrocyte senescence and aging changes in the cartilage matrix. Osteoarthr. Cartil..

[49] Wu K, Laouar L, Dong R, Elliott JAW, Jomha NM (2019). Evaluation of five additives to mitigate toxicity of cryoprotective agents on porcine chondrocytes. Cryobiology.

[50] Hahn J, Laouar L, Elliott JAW, Korbutt GS, Jomha NM (2017). The effect of additive compounds on glycerol-induced damage to human chondrocytes. Cryobiology.

[51] Lemmon, E. W., Huber, M. L. & McLinden, M. O. *NIST Standard Reference Database 23: Reference Fluid Thermodynamic and Transport Properties-REFPROP*. Version 8.0 (National Institute of Standards and Technology, Standard Reference Data Program, Gaithersburg, 2007).

[52] Design Institute for Physical Properties. *DIPPR Project 801—Full Version* (Design Institute for Physical Properties, 2016).

[53] Chen Y (2012). Scratch wound healing assay. Bio-protocol.

[54] Liang Y (2018). Chondrogenic differentiation of synovial fluid mesenchymal stem cells on human meniscus-derived decellularized matrix requires exogenous growth factors. Acta Biomater..

