# Supplementary material for: Vitrification of particulated articular cartilage via calculated protocols
Source: NPJ Regen Med. 2021 Mar 19;6:15. doi: 10.1038/s41536-021-00123-5 (PMC7979917; doi:10.1038/s41536-021-00123-5)
Supplement: Supplementary file 1 — Supplementary material [file 41536_2021_123_MOESM1_ESM.pdf]

Supplementary Materials

Supplementary Table 1: Absolute chondrocyte viability of porcine particulated articular cartilage after vitrification and assessment with a cell membrane integrity stain

Chondrocyte viability (%)	Mean	Standard Deviation	95% Confidence Interval
Positive control	94.2	4.8	[92.0, 96.4]
Negative control	0.1	0.1	[0, 0.1]
Protocol E–D–P: Day 0	84.7	11.0	[79.7, 89.7]
Protocol E–D–P: Day 180	86.5	9.4	[82.2, 90.8]
Protocol E–D: Day 0	85.4	11.6	[80.1, 90.7]
Protocol E–D: Day 180	90.2	5.1	[87.9, 92.6]

Supplementary Table 2: Absolute chondrocyte viability of human particulated articular cartilage after vitrification and assessment with a cell membrane integrity stain

Chondrocyte viability (%)	Mean	Standard Deviation	95% Confidence Interval
Positive control	91.6	4.0	[88.6, 94.6]
Negative control	0.2	0.4	[−0.1, 0.6]
Protocol E–D–P: Day 0	79.1	10.4	[71.1, 87.1]
Protocol E–D–P: Day 180	82.5	11.4	[70.5, 94.5]
Protocol E–D: Day 0	74.7	17.2	[61.4, 87.9]
Protocol E–D: Day 180	76.3	12.1	[63.6, 88.9]

Supplementary Table 3: Relative fluorescence units (mean ± standard deviation) of porcine particulated articular cartilage after vitrification and assessment by alamarBlue

Relative fluorescence units	48 hr	96 hr
Positive control	68663 ± 1946	58913 ± 2497
Negative control	10636 ± 976	15140 ± 1215
Protocol E–D–P: Day 0	72026 ± 2283	68993 ± 3155
Protocol E–D–P: Day 180	71636 ± 3084	67789 ± 5754
Protocol E–D: Day 0	71763 ± 2815	71875 ± 4941
Protocol E–D: Day 180	69620 ± 4030	66602 ± 6100

Supplementary Table 4: Relative fluorescence units (mean ± standard deviation) of human particulated articular cartilage after vitrification and assessment by alamarBlue

Relative fluorescence units	48 hr	96 hr
Positive control	63317 ± 7668	66137 ± 3752
Negative control	5309 ± 2022	9275 ± 1743
Protocol E–D–P: Day 0	56727 ± 5245	72280 ± 11261
Protocol E–D–P: Day 180	54577 ± 221	68845 ± 4047
Protocol E–D: Day 0	53159 ± 9816	69682 ± 5471
Protocol E–D: Day 180	44304 ± 9151	65784 ± 7895
