# Supplementary material for: Vitrification of particulated articular cartilage via calculated protocols
Source: NPJ Regen Med. 2021 Mar 19;6:15. doi: 10.1038/s41536-021-00123-5 (PMC7979917; doi:10.1038/s41536-021-00123-5)
Supplement: Supplementary file 2 — Reporting Summary Checklist [file 41536_2021_123_MOESM2_ESM.pdf]

nature research

Corresponding author(s): Nadr M. Jomha
Janet A.W. Elliott

Last updated by author(s): Jan 7, 2021

Reporting Summary

Nature Research wishes to improve the reproducibility of the work that we publish. This form provides structure for consistency and transparency in reporting. For further information on Nature Research policies, see our Editorial Policies and the Editorial Policy Checklist.

Statistics

For all statistical analyses, confirm that the following items are present in the figure legend, table legend, main text, or Methods section.

n/a	Confirmed	
☐	☒	The exact sample size (*n*) for each experimental group/condition, given as a discrete number and unit of measurement
☐	☒	A statement on whether measurements were taken from distinct samples or whether the same sample was measured repeatedly
☐	☒	The statistical test(s) used AND whether they are one- or two-sided<br>*Only common tests should be described solely by name; describe more complex techniques in the Methods section.*
☐	☒	A description of all covariates tested
☐	☒	A description of any assumptions or corrections, such as tests of normality and adjustment for multiple comparisons
☐	☒	A full description of the statistical parameters including central tendency (e.g. means) or other basic estimates (e.g. regression coefficient) AND variation (e.g. standard deviation) or associated estimates of uncertainty (e.g. confidence intervals)
☐	☒	For null hypothesis testing, the test statistic (e.g. *F*, *t*, *r*) with confidence intervals, effect sizes, degrees of freedom and *P* value noted<br>*Give P values as exact values whenever suitable.*
☒	☐	For Bayesian analysis, information on the choice of priors and Markov chain Monte Carlo settings
☒	☐	For hierarchical and complex designs, identification of the appropriate level for tests and full reporting of outcomes
☒	☐	Estimates of effect sizes (e.g. Cohen's *d*, Pearson's *r*), indicating how they were calculated

Our web collection on statistics for biologists contains articles on many of the points above.

Software and code

Policy information about availability of computer code

Data collection	Matlab, Viability 3.2, Leica LAS-X, CytoFluor II, Image J, SPSS, GraphPad Prism
Data analysis	Matlab was used for model calculation and generate protocol information; A custom made viability software was used to analyze cell viability; Leica LAS-X was used to analyze confocal images of cartilage sample; CytoFluor II was used to detect alamarBlue reaction for chondrocyte metabolic activity; Image J was used to analyze chondrocyte migration; SPSS and GraphPad Prism were used to analyze data and plot figures.

For manuscripts utilizing custom algorithms or software that are central to the research but not yet described in published literature, software must be made available to editors and reviewers. We strongly encourage code deposition in a community repository (e.g. GitHub). See the Nature Research guidelines for submitting code & software for further information.

Data

Policy information about availability of data

All manuscripts must include a data availability statement. This statement should provide the following information, where applicable:

- Accession codes, unique identifiers, or web links for publicly available datasets
- A list of figures that have associated raw data
- A description of any restrictions on data availability

All experimental data were provided in the results or supplemental materials sections. The chemicals and tools required for the experiments were indicated in the methods sections.

Field-specific reporting

Please select the one below that is the best fit for your research. If you are not sure, read the appropriate sections before making your selection.

☒ Life sciences ☐ Behavioural & social sciences ☐ Ecological, evolutionary & environmental sciences

For a reference copy of the document with all sections, see nature.com/documents/nr-reporting-summary-flat.pdf

Life sciences study design

All studies must disclose on these points even when the disclosure is negative.

Sample size	Sample size was chosen based on the study design with minimal of five replicates for animal samples and three replicates of human samples.
Data exclusions	A minimum 80% absolute chondrocyte viability in the positive (fresh) controls before cryoprotectant exposure (chondrocyte viability at t1) was used to screen out unhealthy cartilage donors.
Replication	All attempts at replication were successful.
Randomization	Each cartilage donor was tested for both protocols, no randomization allocated in this study.
Blinding	No blinding was performed in this study and the only criteria of exclusion is a minimum 80% absolute chondrocyte viability in the positive (fresh) controls to screen out unhealthy cartilage donors.

Reporting for specific materials, systems and methods

We require information from authors about some types of materials, experimental systems and methods used in many studies. Here, indicate whether each material, system or method listed is relevant to your study. If you are not sure if a list item applies to your research, read the appropriate section before selecting a response.

Materials & experimental systems

n/a	Involved in the study	
☒	☐	Antibodies
☒	☐	Eukaryotic cell lines
☒	☐	Palaeontology and archaeology
☐	☒	Animals and other organisms
☒	☐	Human research participants
☒	☐	Clinical data
☒	☐	Dual use research of concern

Methods

n/a	Involved in the study	
☒	☐	ChIP-seq
☒	☐	Flow cytometry
☒	☐	MRI-based neuroimaging

Animals and other organisms

Policy information about studies involving animals; ARRIVE guidelines recommended for reporting animal research

Laboratory animals	The study did not involve laboratory animals.
Wild animals	The study did not involve wild animals.
Field-collected samples	Hind legs with joints (N = 23 porcine hind legs) from sexually mature pigs aged over 54 weeks were obtained from a meat-processing plant slaughter house in Wetaskiwin, Alberta, Canada. No animals were specifically euthanized for this research.
Ethics oversight	The Research Ethics Office at the University of Alberta provided ethical approval for the experimental use of animal tissues and cells. Human research ethics approval was obtained from the University of Alberta Research Ethics Office.

Note that full information on the approval of the study protocol must also be provided in the manuscript.
